# Molecular Pathways Involved in Colorectal Cancer: Implications for Disease Behavior and Prevention

**DOI:** 10.3390/ijms140816365

**Published:** 2013-08-07

**Authors:** Dora Colussi, Giovanni Brandi, Franco Bazzoli, Luigi Ricciardiello

**Affiliations:** 1 Department of Medical and Surgical Sciences, University of Bologna, Via Massarenti 9, Pad 5, Bologna 40138, Italy; E-Mails: dora.colussi86@gmail.com (D.C.); franco.bazzoli@unibo.it (F.B.); 2 Department of Experimental, Diagnostic and Specialty Medicine, University of Bologna, Via Massarenti 9, Pad 5, Bologna 40138, Italy; E-Mail: giovanni.brandi@unibo.it

**Keywords:** colorectal cancer, chromosomal instability, microsatellite instability, DNA methylation, aspirin

## Abstract

Research conducted during the past 30 years has increased our understanding of the mechanisms involved in colorectal cancer initiation and development. The findings have demonstrated the existence of at least three pathways: chromosomal instability, microsatellite instability and CpG island methylator phenotype. Importantly, new studies have shown that inflammation and microRNAs contribute to colorectal carcinogenesis. Recent data have demonstrated that several genetic and epigenetic changes are important in determining patient prognosis and survival. Furthermore, some of these mechanisms are related to patients’ response to drugs, such as aspirin, which could be used for both chemoprevention and treatment in specific settings. Thus, in the near future, we could be able to predict disease behavior based on molecular markers found on tumors, and direct the best treatment options for patients.

## 1. Introduction

Colorectal cancer (CRC) is one of the major causes of morbidity and mortality, representing the second major cause of cancer incidence among females and the third among males. Epidemiologists reported that in 2008 the annual worldwide incidence of CRC cases was 1.2 million, almost equally split between males and females [1]. Worldwide incidence appears to be highly variable with increasing trends in countries historically considered at lower risk.

The pathogenesis of CRC is very complex and diverse and is also influenced by multiple factors, some of which are related to diet and lifestyle, while others are related to genetic predisposition. Another risk factor is the presence of long-standing inflammatory bowel diseases (IBD), either Crohn’s or ulcerative colitis [2]. Several epidemiological studies have confirmed the involvement of numerous environmental and dietary factors, such as cigarette smoking, alcohol abuse, a diet high in fat and low in fiber, a sedentary lifestyle and obesity [3]. Physical activity [4], long-term therapy with low-dose aspirin [5], and the Mediterranean diet [6] have proved to have possible preventive effects.

The pathogenesis of CRC varies according to genetic or epigenetic changes, which are related to each other in varying degrees. They follow the multiple stages pattern theorized by Fearon and Vogelstein [7]. Such genetic and epigenetic alterations are directly responsible for a specific event within the sequence that leads to CRC, by contributing to the “initiation” of neoplastic transformation of healthy epithelium and/or determining the “progression” towards more malignant stages of the illness.

The different pathways are characterized by distinctive models of genetic instability, subsequent clinical manifestations, and pathological behavior characteristics. Most CRC follows the chromosomal instability (CIN) pathway, characterized by widespread loss of heterozygosis (LOH) and gross chromosomal abnormalities [8,9]. The second involves approximately 15% of CRC and is due to derangement of the DNA Mismatch Repair (MMR) system and consequential microsatellite instability (MSI). The MMR system is responsible for the production of proteins that recognize and direct repair of single nucleotide mismatches at microsatellite sequences that escape the proofreading system of DNA polymerase.

In recent years, it has been established that other systems and pathways are involved in the pathogenesis of colorectal cancer, including abnormal DNA methylation, inflammation and, more recently the discovery that microRNA (miRNA) can actively contribute to the carcinogenic process. These, along with the aforementioned CIN, MSI and DNA methylation will be discussed.

## 2. CIN Pathway

### 2.1. The WNT Signaling Pathway

CIN is the most well characterized type of colorectal pathway and the most common. The tumorigenic process involves different mitotic spindle checkpoint regulators and proteins that mutually influence mitotic chromosome stability [10,11]. A “key” initial mutation is the early mutation of the adenomatous polyposis coli (APC) tumor suppressor gene, involved in both sporadic CIN and, when germline mutated, in all Familial Adenomatous Polyposis (FAP) [12,13]. In FAP syndrome, an autosomal-dominant genetic disorder characterized by the development of hundreds to thousands adenomas in the colorectum during adolescence and young adulthood, there is a germline mutation of the APC gene that has been identified in 60%–80% of families with FAP [14]. An attenuated form of FAP (AFAP), characterized by less than 100 adenomas, occurs with APC germline mutations involving the 5′ or 3′ region of the gene. Importantly, 16%–40% of patients with less than 100 polyps carry the bi-allelic inactivation of the MUTYH based-excision repair gene, a condition called MUTYH-associated polyposis (MAP). Phenotypically AFAP and MAP are very similar [15].

The APC tumor suppressor gene is involved in APC/β-catenin/Tcf pathway. Its inactivation results in increased WNT pathway signaling, through the failure to degrade β-catenin. The β-catenin cytoplasmic accumulation leads to its translocation into the nucleus and stimulates the TCF-targets, with increased proliferation, differentiation, migration and adhesion of colorectal cells. Mutations in genes implicated in APC/β-catenin/Tcf pathway in CRC lacking APC mutations are also found in sporadic CIN tumors, in particular mutations of β-catenin in 48% of tumors without APC mutations [16], indicating that CTNNB1 mutations are present in the early stages of the colorectal pathogenesis and possibly substitutes the APC mutations in the stages of initiation [16,17].

Also, different components of the WNT/APC/β-cat pathway can be directly or indirectly altered—for example via constitutively activating β-catenin or Tcf. Among the various regulatory genes that interact with the APC suppressor gene, the mitotic checkpoint protein BubR1 was found to play a crucial role. BubR1 is a component of the mitotic checkpoint machinery along with Bub1, Bub3, Mad1, Mad2, Mad3, Mps-1 and CENP-E. By binding to Cdc20 it inhibits APC activity by stimulating a “wait anaphase” signal [18]. Its downregulation and consequent inactivation contribute to the formation of polyploid cells, prolonged cell survival, and excess proliferation, indicating a potential pathogenic mechanism in the initiation of chromosomal instability in CRC sporadic forms [19].

The activity of β-catenin can be indirectly increased by mutations in oncogenes that regulate its activity at various levels. β-Catenin mutually interacts with different members of the Notch pathway, fundamental regulators of cellular differentiation and recently found involved in colorectal carcinogenesis [20]. Kwon and colleagues found that Notch1 increases the accumulation of active β-Catenin protein without needing ligand-receptor activation [21]. They also found that the chronic use of non-steroidal anti-inflammatory drugs (NSAIDs), specifically Ibuprofen, induce a dose-dependent decrease of Notch pathway activity. This confirms the protective effects that have been extensively studied regarding the role of NSAIDs on colorectal cancer [21].

Additional genetic perturbations that modulate β-Catenin activity include *CDK8* (cyclin dependent kinase-8) gene amplification, located at 13q12.13, that is present in approximately 60% of CRC cases. Increased CDK8 kinase activity acts as an oncogene in colorectal cancer by stimulating both β-Catenin [22] and Notch1, thus increasing transcription and cell differentiation [23]. Consistent with previous studies, Firestein *et al.* reported a significant association between *CDK8* expression and β-catenin activation, fatty acid synthase (FASN) overexpression and *p53* expression. *CDK8* over-expression was also significantly correlated with a poor CRC prognosis [24].

Recently, activation of orphan receptors LGR-4 and LGR-5, G-protein-coupled receptors, was found to increase signaling by binding with proteins in the R-respondin family, known potentiators of the WNT signaling pathway. The authors found that the increased Wnt/β-Catenin activity is obtained through enhanced WNT co-receptor LRP6 phosphorylation [25].

Cyclin D1 (CCND1) was also found to be implicated in APC signaling. CCND1, together with other cyclin-dependent kinases that inhibit CCND1, such as *p27* (CDKN1B) and *p21* (CDKN1A), are central to cell cycle control—especially in the transition from G1 to S phase. [26] Excessive CCND1 activation by APC mutation contributes to the development of colonic neoplasia by allowing the cell to escape apoptosis. Arber and colleagues evaluated the presence of CCND1 in normal colonic mucosa, adenoma and adenocarcinoma and confirm its increased expression only in mucosa from individuals affected by CRC [27].

Finally, Morikawa and colleagues - through studies of CRC prevention in obese individuals - found that obesity and physical inactivity increases the risk of developing colorectal cancer without affecting the WNT/beta-catenin pathway [28].

### 2.2. RAS Pathway

The above-mentioned early mutations of CIN pathway, are then followed by subsequent events that promote new mutations and facilitate the tumor’s progression from benign to malignant stages. The adenoma to carcinoma transition is determined firstly by the *K-ras* gene, a proto-oncogene that encodes for the GTPase protein involved in the transduction and propagation of extracellular signals—e.g., mitogen-activated protein kinase (MAPKs). Mutations of *K-ras* lead to a permanently active state that permits the cell to evade apoptosis and acquire a growth advantage. More than 90% of mutations in the *K-ras* gene happen at codon 12 and at codon 13 [29]. Mutations at codon 12 confer a more oncogenic phenotype than the mutations at codon 13, suggesting that codon 13 mutations are more involved in the adenoma-carcinoma transition; whereas codon 12 mutations predispose colorectal tumor cells to local invasion and metastasis [30]. Imamura and colleagues reinforced this hypothesis by confirming that the respective malignancy of the codon 12 and 13 mutations is independent of the *BRAF* mutations that are often associated with a poorer CRC prognosis: even after eliminating *BRAF* as a confounding factor, codon 12 mutations were implicated in significantly higher colorectal cancer specific mortality codon 13 mutations [31]. Studying the mutation in *K-ras* codon 12 and 13, in patients affected by CRC allows for the simultaneous evaluation of CRC prognosis and choice of chemotherapeutic strategies to pursue.

The RAS pathway is also involved with other signals critical for initiation of carcinogenesis. Horst and colleagues demonstrated that high WNT activity was connected with increased MAPK signaling, in *K-ras* mutated CRC samples [32]. Furthermore, Baba and colleagues saw an interaction between the AMP-activated protein kinase (AMPK) and MAPK. AMPK is a cellular energy balance status sensor, and plays a role in the regulation of cell proliferation and growth through the inhibition of the mTOR pathway and activation of the CDKN1A (*p21*) pathway and *p53*. Increased expression of phosphorylated-AMPK is associated with a good prognosis among p-ERK-activated CRC patients [33].

### 2.3. The *p53* System

*p53* loss of function is frequently present in the later stages of colorectal tumorigenesis [34]. The *p53* gene is located on chromosome 17p and its mutation is one of the key steps in colorectal carcinogenesis and stimulates high proliferative activity through the loss of cell cycle control and apoptosis. Oikawa demonstrated that *p53* largely controlled *BubR1* transcription and expression and, in patients with 17p Loss of Heterozygosity (LOH), *BubR1* activity was significantly downregulated [35]. El-Deiry and colleagues identified a wild-type *p53*-activated fragment 1 (WAF-1), a highly induced gene (directly regulated by *p53*) that suppresses tumor cell growth in the *p53* pathway. When *p53* is mutated, the protective role of WAF-1 is not expressed [36]. Another function of *p53* is to regulate energy balance, through activation of the AMPK pathway. Morikawa and colleagues further explored this role of *p53* in energy balance and described that among non-obese patients, *p53* positivity was associated with reduced cancer-specific survival while an adverse effect of obesity on CRC patient mortality was observed in *p53* negative subjects [37].

During the progression of CRC pathogenesis, mutations in different cyclin-dependent kinases (CDKs) are also involved. P53, through the AMPK pathway, up-regulates the CDK inhibitor 1A (*CDKN1A* or *p21*), which is involved in regulating the cell cycle (energy balance status, cellular senescence and stem cell aging). Ogino and colleagues observed *p21* loss of function in 79% of CRC and found this to be significantly associated with *p53* expression. They demonstrated a positive correlation between *p21* loss and CRC survival with increasing patient age, specifically for patients >60 years. Moreover, the adverse effect of obesity in CRC is not observed in *p21* loss CRC [38]. Another CDK associated with *p53* mutations is with CDK inhibitor 1B (CDKN1B or *p27*) [39]. P27 is involved in the control of the progression into S phase of the cell cycle and its degradation is associated with CRC progression [40]. CDKN1B expression is inversely associated with the MSI-H and CIMP-H types of CRC, and more in *p53*-negative cancers [39].

P53 also interacts with Cyclooxygenase-2 (COX-2), which plays a role in promoting inflammation and cell proliferation in CRC [41]. Interestingly, COX-2-positive tumors were found to be associated with an increased cancer-specific mortality regardless of *p53* status, indicating that *COX-2* could be an independent prognostic factor of colorectal cancers [42].

### 2.4. Other Pathways Involved in CIN

An often co-occurring molecular alteration with the *p53* loss is the LOH of chromosome *18q* (65.4%) [43], where the genes *Smad2*, *Smad4* and *DCC* genes are located. LOH of *18q* has been associated with a strong negative prognosis in colon cancer, in particular with high metastatic potential [44]. Ogino studied non-MSI-high CRC tumors associated with *18q* LOH, and found an association with a decreased survival in colorectal cancer patients [45]. In accordance with the previous hypothesis, Lanza and colleagues showed that there was a favorable clinical outcome in negative CRC *18q* allelic loss. They demonstrated that in these patients the 5-years disease-free survival rate was 96% [43].

An alteration that often occurs simultaneously with APC gene mutations is found in the phosphoinositide-3 kinase (PI3KCA) signaling pathway [46], which triggers the activation of different nuclear transcriptional factors through a kinase cascade. Mutations in the *PI3KCA* gene stimulate cell growth as well as the production of fatty acid synthase via the AKT pathway [47]. *PI3KCA* mutations also interact with a central regulator of cell growth and metabolism, *mTOR*, and with *K-ras*. Deming and colleagues demonstrated that the simultaneous presence of *APC* and *PI3KCA* gene mutations in animal models are associated with increased tumor multiplicity and size, and a more aggressive behavior [46]. Finally, Liao and colleagues found that the chronic use of aspirin lengthens survival in individuals with mutated-*PIK3CA* colorectal cancer, possibly by inducing apoptosis via *PIK3CA* inhibition [48].

Recently, it has been hypothesized that LINE-1 (long interspersed nucleotide element-1) extreme hypomethylation is associated with the CIN pathway. LINE-1 (L1) retrotransposon facilitates genomic and chromosomal instability via global DNA hypomethylation and contributes to the expression of non-coding RNA. L1 elements represent approximately 17% of the human genome [49]. Ogino revealed an inverse association between the MSI pathway and LINE-1 hypomethylation [50], and Baba and colleagues also confirmed a direct association between LINE-1 hypomethylation and chromosomal instability (CIN) [51]. Researchers have also found that this pathway presents specific clinical features, characterized by a young age of onset of CRC (<60 years old) and a more aggressive behavior [51,52]. This suggests that LINE-1 extreme hypomethylation may produce a distinct subtype of colorectal cancers with a unique pathogenic mechanism.

Other mechanisms that develop early during tumorigenesis and that contribute to making aneuploidy in the CIN pathway are represented by the alterations of the kinetochore, a multiprotein complex essential for chromosome segregation during mitosis. Centromere protein (CENP)-A and CENP-H are two kinetochore components, whose overexpression was found in a high percentage of CRC; their high expression causes an altered association with non-centromeric regions of chromatin, leading to disruptions of the kinetochore complex [53,54]. Tomonaga *et al.* demonstrated a more prominent induction of aneuploidy by CENP-H overexpression than CENP-A, and a characteristic CENP-H overexpression in CIN colorectal cell lines compared with MSI-H colorectal cell lines [53].

Other components implicated in the CIN pathway include the Hypoxia Inducible Factor (HIF)-1 and HIF-2, which function as essential mediators of cellular response to hypoxia and increase the expression of different genes involved in angiogenesis, cell survival, and glucose metabolism, by influencing different pathways, including *mTOR*. An over-expression of *HIF1α*, a key regulatory subunit of *HIF1* and *HIF2*, has been reported to directly upregulate *COX-2* expression in CRC by binding with *COX-2* [55,56]. Importantly *HIF1α* over-expression was significantly associated with shorter colorectal cancer-specific survival and overall survival [55].

Finally, the expression of cathepsin B (CTSB), a lysosomal cysteine protease, was found elevated in all stages of CRC, from early tumor initiation to metastatic lesions. Chan and colleagues demonstrated that CTSB expression was not associated with CRC stage, but strongly associated with a significant increase in risk of CRC**-**specific mortality and an increase in overall mortality [57].

## 3. MSI Pathway

The MSI pathway represents a form of genomic instability involved in the genesis of approximately 15% of sporadic colorectal cancer and >95% of Hereditary Non Polyposis Colorectal Cancer (HNPCC) syndrome. MSI is caused by the inactivity of the DNA Mismatch Repair (MMR) system. Disabled DNA MMR causes a 100-fold increase in the mutation rate in colorectal mucosa cells [58]. The MMR system is a multi-protein system, which acts like a proofing machine to increase the fidelity of DNA replications by identifications and direct repair of mismatched nucleotides [59,60]. The MMR system acts only when an error eludes the intrinsic error checking system of DNA polymerase [59]. In human cells, the functioning MMR system is composed of multiple interacting proteins including the human MutS homologue (MSH) 2, and human MutL homologue (MLH) 1.

CRC that develops through the MSI pathway presents peculiar clinical features: more often located in the proximal colon, with a poorly differentiated and a mucinous or medullary histotype, and often presents intense peritumoral and intratumoral lymphocytic infiltrations [61]. In general, the prognosis and survival of patients affected by MSI-high CRC is better and longer than that of patients with CIN positive CRC [61]. Importantly, MSI-high CRC does not respond to 5-fluorouracyl-based chemotherapies [62].

In the HNPCC syndrome, CRC development is determined by germline mutation in one of the MMR components. HNPCC is an autosomal dominant genetic disorder characterized by a young age of onset (<50 years old) of colorectal cancer as well as other malignant tumors, including endometrial and ovarian cancers. In 95% of HNPCCs, mutations are present in *hMLH1* and *hMSH2* [63]. The clinical manifestations can be diverse, depending upon which gene is involved and where the mutations occur [64]. Defective *hMSH2* is associated with a 40%–60% increased risk of developing endometrial cancer, while defective *hMLH1* with a 50%–80% increased risk of developing CRC [65,66]. Furthermore mutations in *hMSH6* are associated with 11%–19% increased risk of developing gastric cancer [67] while mutations in *hPMS2* with a 9%–12% increased risk of develop ovarian cancer [68]. Recently, a subclass of the MMR deficient HNPCC families have been found to carry germline deletions of the Epithelial Cell Adhesion Molecule (EpCAM) resulting in *hMSH2* gene silencing [69]. EPCAM carriers show a lower risk of developing endometrial cancers. HNPCC is a good example of a genotype-phenotype association, and the identification of mutation carriers is critical for implementing optimum screening and follow-up procedures [63]. Also, in families with high suspected HNPCC, clinical parameters can help direct new suspected cases toward targeted genetic testing [70].

In sporadic settings, MSI-high CRCs are mostly due to epigenetic silencing of the *hMLH1* gene promoter [71–74]. The resulting mutant phenotype, as in HNPCC settings, leads to inactivation of target genes, in particular tumor suppressors having a microsatellite sequence in their coding region. Importantly, sporadic MSI-high CRC cases harbor the *V600E* mutation of the *BRAF* oncogene, a member of the *RAF* family involved in the mediation of cellular response to the growth signal through the RAS-RAF-MAP kinase [75]. MSI-high sporadic CRCs display CIMP features (a combination of two pathways), and will be described further in the CIMP pathway section.

More than 80% of MSI-CRC harbor mutations of the TGF-β Receptor II (TGF-βRII) [76]. TGF-βRII mutations are found in adenomas either featuring high-grade dysplasia or progressing to adenocarcinoma, and represent a common cause of neoplastic progression in the late and metastatic steps of MSI-High CRCs [77]. Additionally, mutations in the Smad2 and Smad4 genes, part of the TGF-β pathway, are common in MSI-high CRCs [78]. Smad4 mutations facilitate the switch to the tumor-promoting role of TGF-β signaling [79]. Eppert and colleagues demonstrated that the loss of function of Smad2 contributes, independently of Smad4, to deactivated TGF-β signaling [80]. Another mutational target in the genesis of MSI-high CRCs is the alteration of the 2 polyadenine (A8) tracts in exon 10 of the activin type 2 receptor (ACVR2). The ACVR2 gene encodes for a transmembrane receptor, whose activation causes differentiation and growth suppression signaling through the phosphorylation of Smad2 and Smad3 proteins. Jung and colleagues identified these mutations only in MSI-high CRCs—further demonstrating that the *ACVR2* mutation occurred frequently with *TGF-βR2* mutations [81].

Another target gene in the MSI-high CRC pathway is the pro-apoptotic tumor suppressor gene *BAX*. Homozygous frameshift mutations of *BAX* occur in 50% of CRCs cases and promote the cell’s escape from intrinsic apoptosis mechanisms [82,83]. *BAX* gene mutations, like *TGF-βRII* mutations, can be present in neoplastic progressions despite early adenoma mutations [84]. However, Shima and colleagues studied the co-occurring mutations of *TGF-βRII* and *BAX* in a large cohort of patients, and demonstrated that MSI-high CRCs were associated with a better prognosis than MS stable CRCs, regardless of the presence of mutations of *TGF-βRII* and *BAX* [85].

In addition to the above-mentioned genes frequently present in MSI-high CRC, other genes are present at a lower frequency (around 20%) including mutations in the MMR genes *hMSH3* (36.5%) and *hMSH6* (17.5%), Insulin Growth Factor Type 2 Receptor (IGFIIR) (22%), *BLM* gene (16%), PIK3CA (15%), G protein-coupled receptor of Prostaglandin-endoperoxide synthase 2 (PTGS2) (33%) and *Cyclin D1* gene (28%) [86–90].

Recently, Baba and colleagues found that G protein-coupled receptor *PTGER2* overexpression, the downstream target of *PGE2*, which is involved in inflammation and cancer, is strongly associated with MSI [88].

Finally, Ogino demonstrated that the presence of *cyclin D1* in the colon neoplastic mucosa was found not only in patients with the altered CIN pathway, but also in those with the altered MSI pathway. Its overexpression was associated with lower colon cancer–specific, and overall, mortality [91].

## 4. CIMP and the “Serrated” Pathway

A third pathway through which CRC progresses is the CpG island methylator phenotype (CIMP) [92,93]. It consists of the aberrant hypermethylation of CpG dinucleotide sequences localized in the promoter regions of genes involved in cell cycle regulation, apoptosis, angiogenesis, DNA repair, invasion and adhesion. The promoter hypermethylations cause the loss of gene expression. CIMP is found in approximately 20%–30% of CRC and it was reported that clinical features of CIMP CRCs are similar to those associated with MSI [94]. An early event that is correlated with the progression of histologic grades is the silencing of the *p16INK4a* tumor suppressor gene, whose loss of function causes uncontrolled cell proliferation, leading to neoplastic transformation [95–98].

Based on the number of methylated markers, the CIMP phenotype can be also divided into CIMP-high and CIMP-low. The *BRAF* oncogene mutation is often identified in CIMP-high CRC and is associated with increased cell growth, progression of carcinogenesis, and high colon cancer specific mortality [99]. However CIMP-high tumors, regardless of BRAF mutation, are associated with reduced colon cancer mortality [99].

Importantly BRAF V600E mutations were found in 90% of CRC cases with sessile serrated adenoma (SSA) lesions and never in the conventional adenomas. The BRAF mutation is an early event in the serrated pathway and its forced expression will lead to a state of dormancy known as senescence. In SSA, BRAF mutations were found either in early hyperplastic polyps (the serrated precursors) or in the advanced dysplastic serrated polyps, confirming its role in neoplastic progression [100–102]. The SSA polyps and the BRAF mutation frequently have CIMP-high and MSI-high features; thus, researchers established that, in sporadic settings, CIMP-high microsatellite unstable CRCs derive from the serrated pathway [101].

BRAF and KRAS mutations are mutually exclusive [103]. Recently, researchers discovered that when KRAS mutation was found in CIMP CRCs, it is associated with lower markers of methylation, called CIMP-low. This is also frequently associated with mutations in the DNA repair gene Methylguanine Methyltransferase (MGMT) and with the loss of function of the PIK3CA [98,104,105]. CIMP-low, in contrast with CIMP-high, appears to have different phenotype, with a low-level of DNA methylation [106]. An alternative serrated pathway was extensively studied by Jass and colleagues, who described polyps in an “alternative serrated pathway”, as a hybrid of adenomatous and serrated polyps. They hypothesized that these polyps, carrying *K-ras* mutation, represent only 2% of CRC, but present an extremely aggressive malignant potential, through inactivations of MGMT [103,107,108].

The progression out of the senescence state can also be determined by the loss of *p53* function and by the silencing of insulin-like growth factor binding protein 7 (IGFBP7), an important mediator of the *p53*-induced senescence [109]. Ogino S. and colleagues also found that the silencing, and subsequently the downregulation, of cyclin-dependent kinase inhibitor-1B (CDKM1B or *p27*) was associated with CIMP-high CRC and, like IGFBP7, was associated with dysfunctions in *p53* [110]. DNA methyltransferase-3B (DMT3B) overexpression seems to play a role in establishing and maintaining the aformentioned methylation patterns [111].

## 5. Other CRC Pathways

### 5.1. MicroRNA

Recently, microRNAs (miRNAs) have been found to be involved in CRC pathogenesis. miRNA are a class of short (20–22 nucleotide) non-coding RNAs which regulate protein expression by inhibiting mRNA translation, in particular of genes involved in cell differentiation, development, proliferation and apoptosis. The number of miRNAs involved in CRC pathogenesis is very large and still expanding, as new miRNAs are continuously being identified. They can be upregulated or downregulated in CRC, operating like oncogenes and tumor suppressor genes. For example, Bandres and collegues found the altered expression of 13 miRNAs in patients affected by CRC and an interesting, divergent expression of miRNAs in CRCs with either KRAS or BRAF mutations—indicating that these altered expressions may be related to miRNAs’ regulatory action in the RAS pathway [112]. Upregulation of *miR-31* was found to be associated with stage IV CRC [112]. Downregulation of *miR-145* and *miR-143* was demonstrated by other studies, showing that their expression is reduced in precancerous adenomatous polyps, as compared to normal tissue; thus, researchers suggest these miRNAs play a key role in the early development of the tumors [113–115]. Interestingly, Lanza *et al.* found significant upregulations of *miR-17-92*, *miR-17-5p*, *miR-20*, *miR25*, *miR-92-1*, *miR-92-2*, *miR-93-1* and *miR-106a* in the microsatellite stable (MSS) CRC and not in MSI CRC [116]. Furthermore, Motoyama *et al.* demonstrated an increased expression of *miR-31*, *miR-183*, *miR-17-5p*, *miR18a*, *miR-20a* and *miR-92* in tumoral tissue as compared to normal colorectal mucosa, and saw an association between overexpression of *miR-18a* and a worse CRC prognosis [115]. Recently, high expression of *miR-203* was associated with poor survival among Caucasians with stage IV colorectal cancers: and interestingly, it was an indicator of poor survival in blacks with either stages I or II colorectal cancers. Finally, expression of *miR-21* expression predicted a poor prognosis in patients with stage IV cancer [117].

### 5.2. Inflammatory Pathway

Chronic inflammation is a critical component of CRC initiation and progression. This is supported by finding of strong associations between IBD and CRC, and by findings supporting the positive effects of chronic NSAIDs use in CRC. Multiple different markers of inflammation predispose an individual to CRC. This happens by enhancing stimulation, by sustaining cell growth through promoting anti-apoptotic system, and by increasing DNA-damage through the activation of the mutagenic reactive oxygen and nitrogen species. Other mechanisms include the production of angiogenic and lymphangiogenic growth factors, and changes of the membrane systems to facilitate invasion and altering cell adhesion [118]. In support of the role of chronic inflammation in CRCs, researchers studied the role of the pro-inflammatory cytokine tumor necrosis factor (TNF)-α, the transcription factor Signal Transducer and Activator of Transcription 3 (STAT3) protein, Interleukin (IL)-6 and the C-reactive protein (CRP). Chronically elevated levels of TNF-α promote tumor growth, proliferation and metastasis. IL-6 is a cytokine involved in the regulation of the acute phase of inflammation and, in its own transduction pathway, stimulates the transcription of STAT3 [119]. STAT3 activation stimulates its translocation into the nucleus and then stimulates cell proliferation, differentiation, apoptosis and promotes metastasis by inducing the expression of different gene targets—such as *VEGFR2* (vascular endothelial growth factor receptor 2), *Bcl-2*, *CyclinD1*, *MMP2-9*, *ICAM-1*, and *COX-2* [120,121].

CRP is a biomarker of inflammation, both in the acute phase and in the chronic low phase of inflammation [122,123]. The role of this inflammatory mediator was controversial, as researchers obtained discordant results. Chan and colleagues, who investigated the influence of CRP, Interleukin-6 (IL-6) and Soluble Tumor Necrosis Factor Receptor 2 (TNFR-2, a TNF-α receptor superfamily member) in CRC, in a cohort of 33,000 women, found an increased risk of CRC in woman having high levels of sTNFR-2 (*p* = 0.03), but found no correlation with the other two markers [124]. Interestingly, those with high baseline levels of sTNFR-2 who took aspirin had lower risk of developing colorectal cancer. On the other hand Song and colleagues researched the same inflammatory markers, and did not find any correlation, only a positive association between IL-6 and increased risk of CRC in lean individuals (*p* = 0.03) [125]. Moreover, Knupfer and colleagues found higher levels of IL-6 in neoplastic colorectal mucosa than in normal mucosa and strong associations between advanced CRC stage, tumor size and a worse prognosis [126]. Finally, Belluco *et al.* also found a significant association between elevated IL-6 serum levels and worse 5-years survival CRC [127].

Ma and colleagues found an increased level of STAT3 in the abnormal CRC tissue compared with the normal mucosa, and its correlation with CRC metastasis and stage and also its association with cyclin D1 overexpression [120]. However these results were obtained in a very small group of patients.

Interestingly, Otani and colleagues, in a nested case-control study of 38,000 people during an 11 year period, demonstrated CRP to be significantly associated with CRC in the early stages of tumor growth [122], while Gunter found a 25% increase in CRP levels in CRC patients compared to controls, and a stronger association in lean patients [123]. In contrast, in a nested case-control study of 141 patients affected by CRC, no association between CRP levels and the risk of CRC was found [128].

## 6. Conclusions

The findings that different molecular pathways are involved in colorectal cancer development have helped researchers build different models and understand how colorectal cancer initiates and progresses. However, the application of molecular markers on large-scale populations is now facilitating the understanding of the peculiar role of these alterations on disease behavior, prognosis and response to treatments. Among them, the CIMP pathway and the contribution from miRNA require further examination and investigation by researchers for a better and more complete understanding.

The results from the cited studies (Table 1) will be useful for developing strategies, possibly with the use of multiple molecular markers, to predict future disease behavior in newly cancer-diagnosed patients. Importantly, this will help define therapeutic strategies, even with anti-inflammatory drugs, for each individual patient based on their molecular tumor profile. Interestingly, several molecular markers (BRAF and PI3KCA, to cite some) have been found to be predictors of colon cancer risk and mortality in relation to aspirin and anti-inflammatory drugs consumption. As stated above, inflammation is a key contributor to colorectal carcinogenesis and anti-inflammatory drugs have been extensively explored also as chemopreventive agents. The recent findings that long-term use of low-dose Aspirin is protective against colorectal cancer development, clearly indicates that anti-inflammatory drugs could be effectively used to prevent colorectal cancer [5]. However, results from the CAPP-1 [129] and CAPP-2 [130] trials have yielded negative results on the use of aspirin in FAP and HNPCC populations, and the reasons are unclear. Thus selection of patients suitable for chemoprevention should be performed, and possibly baseline inflammatory markers could be of help for the selection process.

## Figures and Tables

**Table 1:** Molecular markers and implications for disease behavior.

Gene	Effect on disease	Ref.
*CDK8* overexpression	Poor prognosis	[24]
*K-ras* cod. 12 mutation	Metastatic disease; poor prognosis; increased cancer specific mortality	[30,31]
*p-AMPK*	Better survival among p-ERK positive	[33]
*p53* expression	Better survival among non obese	[37]
*p21* loss	Better survival for patients >60 yrs	[38]
*COX-2*-positive tumors	Increased cancer specific mortality	[42]
*18q*	Loss in non MSI → decreased survival No loss → 5 year survival 96%	[43,45]
*PI3KCA* mutations	Increased survival among chronic aspirin users	[48]
*Line-1* Hypomethylation	Young age of onset and increased cancer and overall mortality	[50,51]
*HIF1*	High colorectal cancer-specific mortality	[55]
*Cathepsin B* expression	High colorectal cancer and overall mortality	[57]
*MSI*	Better prognosis and survival than CIN/MSS	[61,85,99]
*Cyclin D1* overexpression	Low colon cancer and overall mortality	[91]
*BRAF V600E*	High cancer-specific mortality	[99]
*CIMP*-High	Low colon cancer-specific mortality	[99]
*miR-203*	Poor survival among caucasians with stage IV and poor survival in blacks with stages I and II CRC	[117]
*miR-21*	Poor prognosis in patients with stage IV CRC	[117]
*sTNFR-2* expression	Increased risk of CRC development, lower risk among those taking aspirin	[124]
*Interleukin-6*	Increased risk of CRC development, advanced CRC stage, and a worse prognosis	[125–127]
*C-reactive protein*	Association with increased risk of colorectal cancer, in particular in lean individuals	[122,123]
